# Sustained Intraocular Pressure Reduction Using Bisoprolol-Loaded PLGA Nanoparticles: A Promising Strategy for Enhanced Ocular Delivery with Reduced GFAP Expression Indicative of Lower Glial Activation

**DOI:** 10.3390/pharmaceutics17111418

**Published:** 2025-10-31

**Authors:** Sammar Fathy Elhabal, Omnia Mohamed Mahfouz, Mohamed Fathi Mohamed Elrefai, Mahmoud H. Teaima, Ahmed Abdalla, Mohamed El-Nabarawi

**Affiliations:** 1 Department of Pharmaceutics and Industrial Pharmacy, Faculty of Pharmacy, Modern University for Technology and Information (MTI), Mokattam, Cairo 11571, Egypt; 2 Department of Pharmaceutics and Industrial Pharmacy, Faculty of Pharmacy, Cairo University, Cairo 11562, Egypt; 3 Department of Anatomy and Embryology, Faculty of Medicine, Ain Shams University, Cairo 11591, Egypt; 4 Department of Basic Medical Sciences, Faculty of Medicine, Aqaba Medical Sciences University, Aqaba 77110, Jordan; 5 Pharmaceutics and Pharmaceutical Technology Department, Faculty of Pharmacy, Egyptian Russian University, Cairo 11829, Egypt

**Keywords:** glaucoma, PLGA nanoparticles, bisoprolol, ocular drug delivery, immunohistochemisrty, glial fibrillary acidic protein (GFAP)

## Abstract

**Background/Objectives**: Glaucoma is a neurodegenerative optic disorder which occurs due to persistent elevation of the intraocular pressure. It leads to permanent blindness and currently affects over 75 million individuals worldwide. Nowadays, topical ocular medications are the leading therapy despite their poor ocular penetration and short residence time. **Methods**: The purpose of this research is to formulate bisoprolol hemifumarate-loaded polylactic-co-glycolic acid (PLGA) nanoparticles and improve their ocular penetration and bioavailability for the treatment of glaucoma by enhancing the delivery of the drug to the posterior part of eye. By using the solvent displacement method, formulations were prepared and optimum formula was elected using Design-Expert^®^ software. Results: In vitro characterization demonstrated that the optimum formula contained 25 mg BSP, 22.5 mg PLGA, and 60 mg Tween80, yielding high values of drug encapsulation (75%) and zeta potential (−18.7 ± 0.41 mV), with a low particle size (105 ± 0.35 nm) and polydispersity index (0.411 ± 0.71). Transmission electron microscopy and atomic force microscopy showed smooth and spherical nanosized particles. X-ray diffraction, differential scanning calorimetry, and Fourier-transform infrared spectroscopy revealed successful encapsulation of the drug inside the polymeric matrix. Ex vivo confocal laser scanning microscopy proved that there was better uptake of the drug upon using PLGA-NPs. In vitro release profiles indicated biphasic drug release from the PLGA-NPs, confirming a sustained drug release over 12 h. In vivo studies showed that BSP-PLGA-NPs significantly reduced the IOP compared to bisoprolol solution. Quantitative immunohistochemistry showed lower retinal GFAP expression with BSP-PLGA-NPs compared with induced controls and drug solution, which is indicative of attenuated glial activation. **Conclusions**: These data support improved ocular delivery and an improved pharmacodynamic effect; however, they demonstrate association rather than a direct mechanistic suppression of glial pathways.

## 1. Introduction

Glaucoma is the major cause of sight loss worldwide, as reported by the World Health Organization [1]. It is a severe visual neuropathic disorder marked by the degeneration of retinal ganglion cell axons, abnormalities in the head of optic nerve anatomy, and changes in the retinal nerve fiber layer, which, in turn, leads to permanent blindness [2,3]. Glaucoma occurs due to a persistent increase in intraocular pressure (IOP) (>21 mm Hg) because of a chronic increase in aqueous humor production or a decrease in its outflow [4]. The predominant type of glaucoma is open-angle glaucoma, which is characterized by a resistance toward intraocular fluid outflow within the trabecular mesh despite unobstructed drainage [5]. This condition progresses gradually without pain. If left untreated, the peripheral vision and, subsequently, the central vision are affected, resulting in irreversible blindness. Other uncommon types are closed-angle glaucoma and normotensive glaucoma [6]. The development of closed-angle glaucoma can occur either progressively or unexpectedly [7]. There is a possibility that sudden presentation will be accompanied by symptoms such as intense eye pressure, visual impairments, mid-dilated eye pupils, and eye redness. Eyes that are affected by glaucoma are given the term “glaucomatous” [8].

Current glaucoma therapies seek to regulate the intraocular pressure by reducing aqueous humor production or enhancing its outflow through multiple classes and combinations of topical antihypertensive medicines [9,10]. Treatment is often started by applying a progressive approach, starting with single topical medication therapy, then proceeding to multidrug combinations, and, if necessary, laser treatments and surgery [11]. Topical medication technologies are the most prevalent method for ocular drug delivery [12,13,14]. However, they possess poor ocular penetration to the posterior ocular segment, which leads to low ocular bioavailability of less than 2% and a short residence time, as they encounter significant obstacles including the fast and substantial precorneal loss caused by nasolacrimal elimination, excessive tear flow (tear turn over), ocular barriers (corneal and scleral layers), and insufficient ocular capacity requiring an enhancement in the contact duration between the drug and the corneal surface [15,16,17,18]. Furthermore, they need multiple installations to accomplish the aimed therapeutic effect, which may lead to unfavorable adverse effects due to systemic drug absorption [19,20]. Conventional formulations like Timoptic, Trusopt, and Azopt are administered three to four times daily. However, such frequent dosing may affect patient adherence to treatment, as mentioned before by [4,13]. Extended eye contact makes topical ocular formulations like Xalatan, Lumigan, and Vyzulta among the most effective, as they enhance the drug concentration in the cornea and promote effective drug release [21]. They conquer aspects like enzyme activity and pH, which in turn influence gastrointestinal drug absorption, facilitating sustained-release distribution over multiple days, which is advantageous over short-lived medicines with a brief elimination half-life [22,23].

Reducing the intraocular pressure has been shown to slow down the visual field’s degradation, preventing the onset and progression of glaucoma [24,25]. IOP-lowering agents are first-line medications used in glaucoma treatment [26,27]. They are categorized into two major classes: agents that lower aqueous humour production like Beta-blockers such as timolol, betaxolol, nebivolol, and bisoprolol, carbonic anhydrase inhibitors such as brinzolamide and dorzolamide, and agents that elevate aqueous humour drainage like prostaglandins, analogous to latanoprost, bimatoprost, and Rho-kinase inhibitors such as netarsudil [28,29,30].

Beta-blockers are the first choice of glaucoma treatment. Bisoprolol hemifumarate is a selective beta-adrenergic receptor blocker that lowers aqueous fluid synthesis by blocking β1 receptors to antagonize catecholamine action, as epinephrine stimulates cAMP synthesis and suppresses intraocular fluid production [31,32]. In contrast to non-selective β-receptor antagonists, BSP has fewer systemic side effects in patients who are asthmatic and offers extra neurologically protective effects through scavenging free radicals, lowering inflammation, and reducing excitotoxicity by inhibiting calcium channels and glutamate release. Certain beta-blockers may also have antioxidant attributes [33,34,35].

Unfortunately, its high molecular weight (767 g/mol) and hydrophilic nature (logP value 2.2) limit its ocular penetrability and bioavailability [36,37]. Nanotechnological medication delivery systems are viable for encapsulating active hydrophilic and lipophilic chemicals and delivering them to target areas [38,39]. The development of ocular drug delivery via a nanotechnology system (nano-scale carriers < 1000 nm) like niosomes, micelles, and different polymeric vesicles was significant for managing eye disorders [31,40]. Controlled medication administration, particularly at the ophthalmic level, offers substantial advantages, such as improving therapeutic efficacy, enhancing patient compliance, and minimizing adverse effects [32].

Polylactic-co-glycolic acid (PLGA) is a biocompatible, biodegradable, and non-toxic polymeric nanoparticle that offers promising prospects for prolonged ophthalmic medication delivery applications [41,42,43]. PLGA is randomly degraded into biocompatible metabolites, lactic acid, and glycolic acid through hydrolysis without any enzymatic activity, as mentioned in most of the literature, then removed from the body as water and carbon dioxide [44,45]. The manufacturing techniques of several drug-loaded biodegradable devices that cause the polymer to have numerous advantages as a carrier, including protection of the drug from enzymatic inactivation by tear film or corneal tissues, enhancement of its corneal penetration, precorneal region stability, and minimization of adverse effects on other body organs, were discussed by [46,47].

PLGA is a common biodegradable excipient in authorized drug delivery systems. In the United States, PLGA is listed in the FDA’s Inactive Ingredient Database and on the labels of approved products. In the European Union, Ozurdex is authorized by the EMA as a biodegradable intravitreal implant, which demonstrates regulatory approval of PLGA-based delivery methods [48]. Also, unlike the natural polymers, these synthetic polymers exhibit precise reproducibility and control the degradation rate of those nano systems. A PLGA-based formulation is used for treating occlusive vascular disease and diabetic retinopathy, which confirms the ocular stability of PLGA. PLGA nanoparticles have demonstrated significant stability with prolonged drug release in several trials [49,50,51].

To our best knowledge, this is the first research that has developed bisoprolol hemifumarate nanoparticles using polylactic-co-glycolic acid, a biodegradable polymer and hypotensive agent, to prolong the duration of drug stay on the corneal surface, improve drug bioavailability, promote drug delivery to the back segment of the eye, and enhance patient. The optimum formulation was evaluated in terms of its particle size, size distribution, zeta potential, and encapsulation efficiency using microscopic examination and analysis by Fourier transform-infrared spectroscopy, differential scanning calorimetry, and atomic force microscopy. Furthermore, the dialysis membrane was also used to determine the drug release in vitro. An ex vivo study was conducted using confocal laser scanning microscopy analysis. Histological analysis, the intraocular pressure dynamical effect, and the GFAP marker by immunohistochemical examination were used to evaluate the antiglaucoma activity impacts in the dexamethasone-induced glaucoma rabbit model.

## 2. Materials and Methods

### 2.1. Materials

Bisoprolol hemifumarate was provided as a gift from Global Napi Pharmaceuticals on 6th October, in the industrial zone of Cairo. PLGA; poly (D, L-lactic-co-glycolic acid) ester terminated (430471) with molecular weights of 50,000–75,000 (g/mol), inherent viscosity (IV) 0.55–0.75 (dL/g), and composition ratio 85:15 were purchased from Sigma-Aldrich (Dorset, UK) and stored at temperature of 2–8 °C. Acetone, Tween 80 (poly sorbate 80), dialysis bag cut off 12,000–14,000 Da, was obtained from Sigma-Aldrich. Water filtered via the Millipore^®^ MilliQ system was utilized for all experimental trials. All chemicals and solvents were of high-grade analytical purity.

### 2.2. Methods

#### 2.2.1. High-Performance Liquid Chromatography Analysis for Bisoprolol Hemifumarate

High-performance liquid chromatography analysis on BSP samples was done with an Agilent 1260 series (Agilent Technologies Inc., Santa Clara, CA, USA). An Agilent (C18 column 4.6 × 250 mm, 5 µm; 20 µL injection; 1.2 mL min^−1^; 225 nm; 25 °C) from Phenomenex, Torrance, CA, USA, was used to sort the sample components. The mobile phase was acetonitrile/phosphate buffer (10 mM KH_2_PO_4_, pH 4.5) at 50:50 (*v*/*v*), run isostatically. After a 20 μL sample was placed in the solution, chromatography was done with a flow rate of 1.2 mL/min and a retention period of five minutes. At 225 nm, a UV monitor with a column temperature of 25 °C was used to calculate BSP concentration. This was performed using a standard calibration curve with a range of 10–50 μg/mL and a correlation coefficient of R^2^ > 0.999 [52,53].

#### 2.2.2. Formulation of Bisoprolol Hemifumarate-Loaded PLGA Nanoparticles

Sixteen formulations of BSP-PLGA-NPs were prepared using the solvent displacement (nanoprecipitation) method. In brief, this process involved the development of two separate phases, an organic phase (drug/polymer solution) and an aqueous phase (stabilizer solution) to promote spontaneous nanoparticle formation upon interfacial diffusion. Initially, the organic phase, different weighed amounts of PLGA, and bisoprolol were dissolved in 5 mL of acetone using gentle magnetic stirring until a clear solution was obtained. Then, aqueous phase was prepared by dissolving polysorbate 80 (Tween 80) at different concentrations in 10 mL of Milli-Q^®^ (MilliporeSigma, Burlington, MA, USA) water and maintaining the solution at 25 °C. The organic phase was then added dropwise (1 mL/min) into 10 mL of the aqueous phase under constant stirring at 500 rpm for 5 min using a magnetic stirrer; this procedure was conducted at 25 °C. Subsequently, under reduced pressure, acetone was eliminated from the formulation utilizing a rotary evaporator [54,55,56].

#### 2.2.3. Experimental Design

The BSP-loaded PLGA nanoparticles were formulated using a multilevel factorial design consisting of 16 formulations, from which the optimal formula was selected for further investigation. The parameters examined were bisoprolol (X1), PLGA (X2), and Tween 80 (X3), each evaluated at three levels designated as (−1, 0, +1). This study examined particle size (PS) (Y1), polydispersity index (PDI) (Y2), zeta potential (ZP) (Y3), and entrapment efficiency percentage (EE %) (Y4) as response parameters by applying Design Expert^®^ version 13 (Stat Ease, Inc., Minneapolis, MN, USA) to assess the significance of the parameters under investigation. Table 1 depicts the factorial design, including the levels of the independent parameters and the boundaries applied to the response of corresponding parameters as well.

#### 2.2.4. Physicochemical Characterization of BSP-PLGA Nanoparticle Formulations

##### Particle Size (PS) and Polydispersity Index (PDI), Zeta Potential Determination

- *Particle size and Polydispersity Index*

Dynamic light scattering (HORIBA, SZ-100, Kyoto, Japan) was used to assess the particle size (PS) and the polydispersity index (PDI) of the nanosuspensions. PDI value indicates the uniformity and distribution of particle sizes within the preparation Exactly 0.1 mL from each prepared BSP-PLGA nanoparticle was diluted with 10 mL of MilliQ^®^ water before the measurements. The produced aqueous dispersion was then sonicated using a bath sonicator for 2 min. All experiments were performed at room temperature three times [57,58]. We employed the Z-average (cumulants); intensity-weighted distributions are primary; all experiments were conducted in triplicate (*n* = 3).

- *Zeta potential*

The measurements were conducted with the Zetasizer Nano ZS (Malvern Instruments, Malvern, UK) at 25 °C. The surface charge of the particles was assessed using ZP and quantified through laser-Doppler electrophoresis with the M3 PALS system in a Zetasizer Nano ZS (Malvern Instruments, Malvern, UK), after 100-fold dilution in deionized water [59,60].

- *Entrapment Efficiency Determination*

Entrapment efficiency (EE%) of BSP-NPs was calculated indirectly by estimating free BSP (unentrapped BSP). A total of 1 mL of the resultant formula was centrifuged for one hour at 25,000 rpm and 4 °C, using a Beckman Optima^®^ Ultracentrifuge (Brea, CA, USA). EE% indicates the entrapment efficacy, which is determined by the total quantity of bisoprolol to the quantity of unentrapped (free) drug. The nanoparticles that were sedimented at the base of the centrifuge tube were washed using 7.4 pH phosphate buffer and subsequently underwent a second centrifugation to remove any unentrapped drug [61,62]. The nanoparticles were washed three times to ensure the complete removal of any unencapsulated drug. The purified nanoparticles were then preserved for later characterization, and the concentrations of unentrapped BSP were measured after isolating the clear supernatant using the HPLC method as mentioned previously [63,64].

(1) $$EE(\%)=\frac{total\;quantity\;of\;BSP-free\;quantity\;of\;BSP}{total\;quantity\;of\;BSP}\times 100$$

##### Optimization of Bisoprolol Hemifumarate Loaded with PLGA Nanoparticles

Numerical optimization was employed using Design-Expert^®^ software version 13 (Stat-Ease, Inc., Minneapolis, MN, USA) to identify desirable factors while excluding non-significant ones. The highest values of EE% and ZP, and the lowest values of PS and PDI, were selected as shown in Table 2. The most optimal formula was selected for further assessment.

#### 2.2.5. In-Vitro Characterization of the Optimum Formula

##### Transmission Electron Microscopy

Transmission electron microscopy is a method for visualizing a sample of nanoparticles using an electron beam. TEM is the preferable technique to obtain precise measurements of nanoparticle size, size distribution, and morphology. The optimum BSP-NPs formula surface morphology was examined using a transmission electron microscope (TEM) (JEM-1230, Joel, Tokyo, Japan). Samples were stained negatively with a 1% phosphotungstic acid (PTA) adjusted to neutral PH with sodium hydroxide NOAH and put on a carbon-coated grid surface before being dried for investigation at ambient temperature [65,66].

##### Atomic Force Microscopy

Atomic force microscopy was applied using a scanning probe microscope (Shimadzu, Kyoto, Japan) to evaluate the roughness and morphology of the surface. A glass slide affixed with BSP-NPs was scanned using tapping mode (2 m scan size, 0.894 Hz scan rate, 512 samples per line) [67].

##### Fourier-Transformed Infrared Spectroscopy

Fourier-transformed infrared spectroscopy was employed to examine drug–polymer interactions; the FTIR spectra for 3 mg samples of the pure drug BSP, freeze-dried BSP-NPS, and a blank were measured using the potassium bromide (KBr) disc method on a PerkinElmer Spectrum BX FTIR (PerkinElmer, Waltham, MA, USA). A spectral range of 4000–400 cm^−1^ with a resolution of 4 cm^−1^ was employed to measure the % transmittance [61].

##### Differential Scanning Calorimetry

The simultaneous thermal analyzer STA 4000 (Perkin Elmer, Waltham, MA, USA) was applied to perform differential scanning calorimetry analysis. Samples (3 mg) of the pure drug (BSP), freeze-dried BSP-NPs, and a blank were analyzed at a heating rate of 10 °C/min from 10 °C to 400 °C, with a flow rate of nitrogen at 10 mL/min [65].

##### X-Ray Diffraction Spectroscopy

An X-ray diffractometer (Rigaku MiniFlex 600, Tokyo, Japan) was used with Ni filtered at a generator voltage of 40 kV and a tube current of 40 mA. X-ray diffraction spectroscopy was carried out on pure BSP, freeze-dried BSP-NPs, PLGA, and Tween 80 using Cu Kα radiation. Samples were scanned from 3 °C to 80 °C at a rate of 1° min^−1^ [15].

##### In-Vitro Drug Release Analysis

The in vitro release of bisoprolol from BSP-NPs was assessed using a dialysis bag method. The dialysis membrane (with a standard molecular weight limit of 14,000 Da; Sigma-Aldrich Co.) was immersed in BSP and was assessed using a dialysis bag method in simulated tear fluid (STF, pH 7.4) composed of 0.680 g NaCl, 0.220 g NaHCO_3_, 0.008 g CaCl_2_·2H_2_O, and 0.14 g KCl per 100 mL of distilled water [68]. Dialysis bags containing 2 mL of BSP solution and BSP-NP suspension were put in a 25 mL release medium within dark bottles. The bottles were subsequently promoted in a shaker operating at 37 ± 0.5 °C and 100 rpm. To preserve sink conditions, 3 mL samples were taken at specified intervals (0, 0.5, 1, 2, 4, 6, 8, 10, 12, 24, and 48 h) and replaced with an equal amount of a fresh medium [42,69]. The samples were analyzed, and the released % was calculated using the HPLC method as previously described. The kinetics of BSP release from all BSP-NP formulations were analyzed by linear regression to determine their fitting to either zero-order kinetics, first-order kinetics, or the Higuchi diffusion model. The correlation coefficient (R^2^) was determined for every model and the highest value of R^2^ indicated the best-fit model to the mathematical equation.

#### 2.2.6. Short-Term Stability of Bisoprolol Hemifumarate Loaded with PLGA Nanoparticles

The optimum formula nanoparticles were stored at 25 °C and 4 °C for three months. Afterwards, samples were collected and the meaning of ZP, PS (examined by a Zeta-sizer Nano ZS from Malvern Instruments Ltd., Malvern, UK) and EE% was evaluated. The experiments were conducted three times [70,71].

#### 2.2.7. Ex-Vivo Analysis

##### Confocal Laser Scanning Microscopy Analysis

The formulae were prepared as previously described, then we added 1% (*w*/*v*) rhodamine B (RhB) together with BSP-NPs. Determining the optimum BSP-NP infiltration throughout the cornea’s several layers was the aim of this investigation. To duplicate the application of the optimum formulae in touch with the eye’s surface, BSP-NPs were administered to the cornea’s surface [72,73]. The corneal tissue’s fluorescence was recognized by cutting longitudinal slices sealed in paraffin wax into many sections using a microtome (Rotary Leica RM2245; Leica Biosystems, Wetzlar, Germany). Slides were investigated under an inverted microscope (LSM 710; Carl Zeiss, Oberkochen, Germany). The RhB’s excitation and emission had respective maximum wavelengths of 545 nm and 560 nm. LMS Image provided confocal images. Released in Jena, Germany, version 4.2, the Carl Zeiss Microimaging GmbH browser program was applied.

### 2.3. In Vivo Analysis

#### 2.3.1. Animals

Four groups of albino male rabbits (weighing 2 ± 0.5 kg), with each group containing 6 rabbits, were kept in shelters at 25 °C and 50% relative humidity. They were acclimated for seven days before any clinical trials were performed, during which they were provided with their regular food and drink. The rabbits were kept at 25 °C with a 12 h day/night cycle and unrestricted food and drink access. The Ethics Committee sanctioned the tests, which were conducted following the Research Ethics Committee’s rules (PI 2984).

#### 2.3.2. Ocular Irritancy Test (Draize Test)

The Draize test was used, with the aim of determining whether the formulation was safe to use or not. The evaluation of probable visual irritation and/or undesirable effects of the optimized formulation was performed by observing allergy, irritation, or increased lacrimal secretion following application to albino rabbits’ eyes. Three albino rabbits were utilized in the experiment. The rabbits received, daily, two drops (100 μL) of the tested BSP-PLGA-NPs (10 mg/mL equivalent to 1% BSP) in the left eye at intervals of 0, 1, 2, 3, 4, 5, 6, 24, 48, 72 h, 7, 14, and 21 days while the right eye was treated with saline and kept as a control. Scores on the Draize test range from 0 (no irritation) to 3 (maximum irritation and redness) [32,74,75].

#### 2.3.3. Induction of Glaucoma by Using a Glucocorticoid

**Figure 1:**
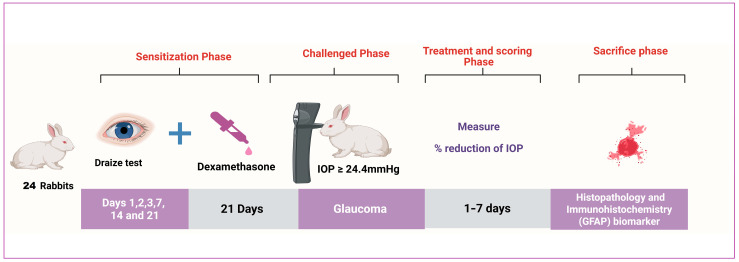
Graphic of in vivo experimental design.

One effective synthetic glucocorticoid that can cause open-angle glaucoma is dexamethasone [76,77]. It causes ocular hypertension (OHT) and primary open-angle glaucoma (POAG) due to increased aqueous humor outflow resistance. Topical dexamethasone eye drops (0.1%) were added to the rabbits’ right eye three times daily for 21 days, then the IOP progression was assessed to ensure ocular hypertension induction. The model was deemed successful if the intraocular pressure (IOP) exceeded the usual upper limit of 24.4 mmHg and persisted for a week. IOP was measured by Tonovet^®^ tonometer (Vantaa, Finland). Each group had 6 rabbits randomly classified as the normal group a (negative control; no dexamethasone induction; no treatment (right eye untreated; left eye saline for Draize)), Group b (left untreated (positive control), dexamethasone-induced ocular hypertension in the right eye, no antiglaucoma treatment (right eye serves as disease comparator; left eye saline for Draize), Group c (treated with bisoprolol solution (dexamethasone induction in the right eye, followed by topical bisoprolol solution per protocol; left eye saline for Draize)), and Group d (treated with BSP-PLGA-NPs (dexamethasone induction in the right eye, followed by topical BSP-PLGA nanoparticles per protocol; left eye saline for Draize)) [78,79]. Every rabbit’s left eye served as a control group while their right eye was treated independently using every formula. For seven days, the intraocular pressure was monitored; results of lowering the IOP were then compared as shown in Figure 1. The process was carried out, with a week of washing separating each time. The % decrease in IOP can be obtained using the equation presented below.

(2) $$\%\mathrm{Decrease}\;\mathrm{in}\;\mathrm{IOP}=\frac{IOP\;Control\;eye-IOP\;dosed\;eye}{IOP\;control\;eye}\times 100$$

### 2.4. Histopathological Examination

Ciliary bodies, retinas, and corneas from deceased animals were extracted and dispatched to the pathological department in a 10% formalin solution under stringent sterile conditions to assess the glaucomatous eye and the efficacy of the ideal treatment formula for this medical disease. Before fixation, the crystalline lens and cornea were excised from each eye, leaving only the eye cups intact. After standard gradual ethanol evaporation, specimens were submerged in xylene at 60 °C for 10 to 15 min. Specimens were subsequently immersed in paraffin wax at 60 °C for 6 h. Retinas were sectioned at a thickness of 5 μm along the sagittal axis, parallel to the optic nerve. Finally, hematoxylin and eosin were utilized to stain the slides (H&E). By using a light microscope, all H&E-stained slides were analyzed to evaluate the severity of the glaucoma’s characteristics in various regions of the eye [15,80].

### 2.5. Immuno-Histochemistry

IHC is used to identify inflammatory markers like GFAP, Iba1, Brn3a, and RGC survival in neurological disorders, particularly those affecting the retina, such as glaucoma. Glial fibrillary acidic protein (GFAP) is a major marker found in Müller retinal cells [81]. GFAP expressions are measured using immunohistochemistry in a glaucoma rabbit model. In glaucomatous eyes, the retinas probably exhibit altered astrocyte morphology and elevated GFAP expression. This would be in line with how neuroinflammation and astrocyte activation contribute to the development of glaucoma [82,83]. In this manuscript, we measure expression levels of GFAP marker. Eye sections were placed and cut on adhesive slides, then the paraffin wax was removed, and the slides were re-hydrated with distilled water; afterward, a heat-induced epitope retrieval step was conducted, and tissue sections were incubated with primary anti-GFAP (Abbexa, Cambridge, UK) for an hour at 25 °C. After washing, the HRP-labelled detection kit (BioSB, Santa Barbara, CA, USA) was used following manufacturer’s instructions to develop the color. Negative control slides were attained by escaping incubation with primary antibodies. Positive expression was quantified as the mean area percentage in five random high-power microscopic fields representing each group [84,85].

### 2.6. Statistical Analysis

The statistical analysis was applied with Design-Expert^®^ software. A one-way analysis of variance was carried out to compare groups. Differences were assessed as statistically significant when the *p*-value was <0.05. The experimental data were illustrated as mean ± standard error of the mean.

## 3. Results and Discussion

### 3.1. Physicochemical Characterization of Bisoprolol Hemifumarate Loaded with PLGA Nanoparticles

#### 3.1.1. Particle Size, Polydispersity Index, Zeta Potential and Entrapment Efficiency Analysis

**Figure 2:**
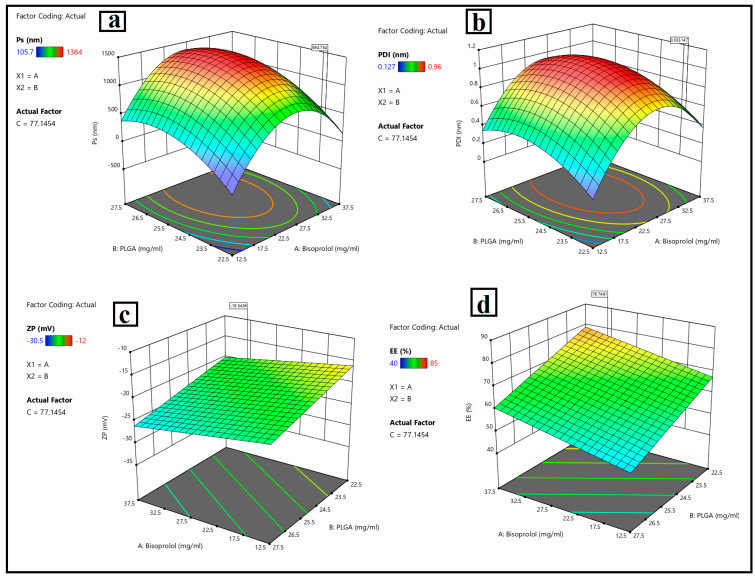
Response 3D plots for the effect of (A): bisoprolol hemi fumarate amount, (B): PLGA amount, and (C): Tween 80 amount, on (**a**) particle size, (**b**), polydispersity index, (**c**) zeta potential, and (**d**) entrapment efficiency %.

The pertinent physicochemical properties of the nanoparticles were illustrated in Table 2, Figure 2a–d, which indicates the response variables and surface study findings.

- *Particle size analysis*

The size of the particles is a fundamental factor for drug delivery systems that are based on nanoparticles. The size of a nanoparticle might range from 10 nm to 1000 nm for delivery purposes. It is one of the factors which control the kinetics of drug release, as smaller particles will be related to faster drug release, cellular uptake, and biodistribution and higher stability. References [86,87] have demonstrated that nanoparticle penetration into the corneal layers is mostly based on the particle size.

Figure 2a depicts that, when the drug (bisoprolol) amount (Y1) increased from 12.5 mg to 37.5 mg, the particle size of BSP-loaded PLGA nanoparticles (Y1) increased from 105 ± 0.35 nm to 784 ± 0.71 nm due to the encapsulation of the drug inside the polymeric matrix of the nanoparticles Regarding the PLGA amount (Y2), when the PLGA amount increased from 22.5 mg to 27.5 mg, the particle size of the nanoparticles was increased, which contributed to an increase in the dispersion viscosity, leading to particle agglomeration, as previously explained by [4] in the formulation of dorzolamide-loaded poly D, L-lactide-co-glycolic nanoparticles. Reference [88] also noticed an increase in particle size with increases in the PLGA amount, which complied with the result stated by [89], who explained that the increase in particle size of the nanoparticles is due to increasing the amount of PLGA, owing to it reducing the net shear stress and favoring the production of bigger particles. Furthermore, the increased viscosity may hinder the rapid dispersion of the PLGA solution into the aqueous phase, forming bigger particles, which then produce larger nanoparticles following the removal of the organic solvent. When increasing the amount of surfactant (Tween 80) (Y3) from 60 mg to 100 mg, the particle size of the nanoparticles decreased. These findings may contribute to the stabilization effect of the surfactant during nanoparticle production and reduce surface energy, inhibiting crystal development. In contrast, the experiment by [90] found that, upon increasing the amount of polysorbate 80, which functioned as an amphiphilic compound, the particle size increased due to its adsorption on the particle’s surface [91,92]. Run 9 has a PS (105 ± 0.35 nm) that is ideal for corneal penetration and ocular retention while avoiding irritation (ocular penetration strongly depends on sub-200 nm size).

- *Polydispersity index assessment*

The polydispersity index is an essential parameter for characterizing the fluctuation in particle size within a nano system. When the polydispersity index approaches 1, the size range expands. The polydispersity index’s ideal value is near zero, which is indicative of a monodispersed system. This parameter is directly related to dispersion stability, which indicates stable nano systems. Figure 2b shows that the response (Y2) ranged from a minimum of 0.22 nm to a maximum of 0.96 nm. When the amount of drug (Y1) increased, the PDI values increased due to encapsulating the drug inside the nanoparticles. Regarding increasing the amount of PLGA (Y2), PDI values were increased due to increasing the viscosity of the dispersion, as mentioned before. Increasing the surfactant amount (Y3) reduces the interfacial tension, resulting in a reduction in particle size and hence increasing the degree of homogeneity, and the same results were obtained by [93]. Run 9 has a PDI ≈ 0.411 (unitless). Although broader than ~0.2–0.3, it is still serviceable for PLGA systems produced by nanoprecipitation, especially at the lowest stabilizer level (Tween 80 = 60 mg) during the optimization and characterization of brimonidine tartrate nanoparticles loaded in in situ gel for the treatment of glaucoma.

- *Zeta potential assessment*

The value of the zeta potential is an essential aspect regarding the stability of the colloidal system. NPs with zeta potential values < −30 mV or >+30 mV demonstrated improved stable dispersion. Some nanoparticles exhibit reduced zeta potential interactions due to Van Der Waals interparticle interactions. Moreover, optimal repulsion between the same charged particles prevents aggregation and ensures dispersibility as well [94,95]. Bisoprolol hemi-fumarate-loaded PLGA nanoparticle formulations showed a negative zeta potential value ranging from −13.3 mv to −30.5 mv, which suggests that free drugs are externally localized and adsorbed on the surface of polymeric nanoparticles. Higher negative zeta potential values limit particle aggregation through the steric hindrance effect, and smaller particle sizes may enhance the effective absorption of drug-loaded nanoparticles in biological systems, as shown in Figure 2c. Sufficient colloidal stability was measured by ZP for Run 9 (−18.7 ± 0.41 mV). While less negative than −30 mV, the system uses nonionic Tween 80; steric stabilization complements electrostatic repulsion, which is consistent with stable dispersions observed pre-storage and the short-term stability trend reported.

Figure 2c showed that ZP values of BSP-PLGA-NPs (Y3) were slightly increased by increasing the amount of drug (X1), because of deprotonation (H+) of the carboxylic group existing in the fumaric acid giving a negatively charged moiety. Upon increasing the PLGA amount (X2), ZP negative values of NPs were increased. This was owing to the existence of carboxylic acid groups inside the lactic acid and glycolic acid of PLGA that deprotonated at physiological pH. Negatively charged systems are believed to be less harmful than positively charged NPs, which can cause cell lysis by breaking the membrane, as stated previously by [96] in the development and characterization of nanoparticle-laden hydrogels for sustained ocular delivery of norfloxacin in the treatment of pseudomonas keratitis and by [20] upon the development and optimization of vitamin E TPGS-based PLGA nanoparticles for improved and safe ocular delivery of ketorolac. It was observed that the negative charges decreased when increasing the amount of Tween 80. The nanosystem was negatively charged due to the presence of a carboxylic group of PLGA. By increasing the Tween 80 amount, the system would be neutralized and hence become lowly negatively charged. This would affect its stability. Therefore, a low amount of Tween 80 could provide an ideal negatively stable system.

- *Entrapment Efficiency assessment*

Entrapment efficiency (EE) measures the nanocarrier’s ability to encapsulate the drug. As depicted in Figure 2d, the impact of the independent variables, drug amount (X1), PLGA amount (X2), and Tween 80 amounts (Y3) on the response (Y4) EE% of BSP-PLGA-NPs ranged from 40–85%. It was found that the EE% increased when increasing the amount of the drug (X1) from 12.5 mg to 37.5 mg, owing to the encapsulation of the drug inside the PLGA nanoparticles. Regarding the PLGA amount (X2), upon increasing the PLGA amount from 22.5 mg to 27.5 mg, it was observed that the EE% tended to increase when increasing the PLGA amount to a certain level, after which we observed a decrease in encapsulation of the drug. This could be attributed to the hydrophilicity of BSP, which might favor the leakage of the drug from the polymer’s lipophilic core to the external aqueous phase. Similar results were obtained by [97] when they studied the continuous delivery of propranolol from liposomes-in-microspheres to inhibit infantile hemangioma growth. Increasing the Tween 80 amount (Y3) reduces the interfacial tension between particles, leading to particle size reduction, which in turn reduces the amount of drug encapsulated inside the NPs and hence promotes faster drug release. Good encapsulation (EE%) was observed for Run 9 (75 ± 0.98%), clearly higher than most alternative small-PS runs (e.g., Runs 3/4/15 at 140–147 nm have 55–65% EE). Consequently, the formulations comprising a low Tween 80 level revealed high EE%, and the same results were obtained before by [98] in the optimization of propranolol-loaded trehalosome.

#### 3.1.2. Optimization of BSP-PLGA Nanoparticles

The design expert software evaluated the formulas to determine the one with the optimal values for particle size, polydispersity index, stable ZP, and maximum EE%. The formula found to be the most appropriate was designated as Run 9. It consisted of 25 mg BSP, 22.5 mg PLGA, and 60 mg Tween 80, as detailed in Table 2. The optimum formula showed the lowest PS value of 105.7 nm, PDI value of 0.411 nm, stable ZP value of −18.7 mv, and high EE% value of 75%. Run 9’s conditions (lower Tween 80, 60 mg), where the stabilizer is at the minimal level among the low-PS runs, predisposed it to mild heterogeneity during nanoprecipitation/solvent removal and were selected for further studies.

### 3.2. In Vitro Characterization of the Optimum Formula

#### 3.2.1. Transmission Electron Microscopy

**Figure 3:**
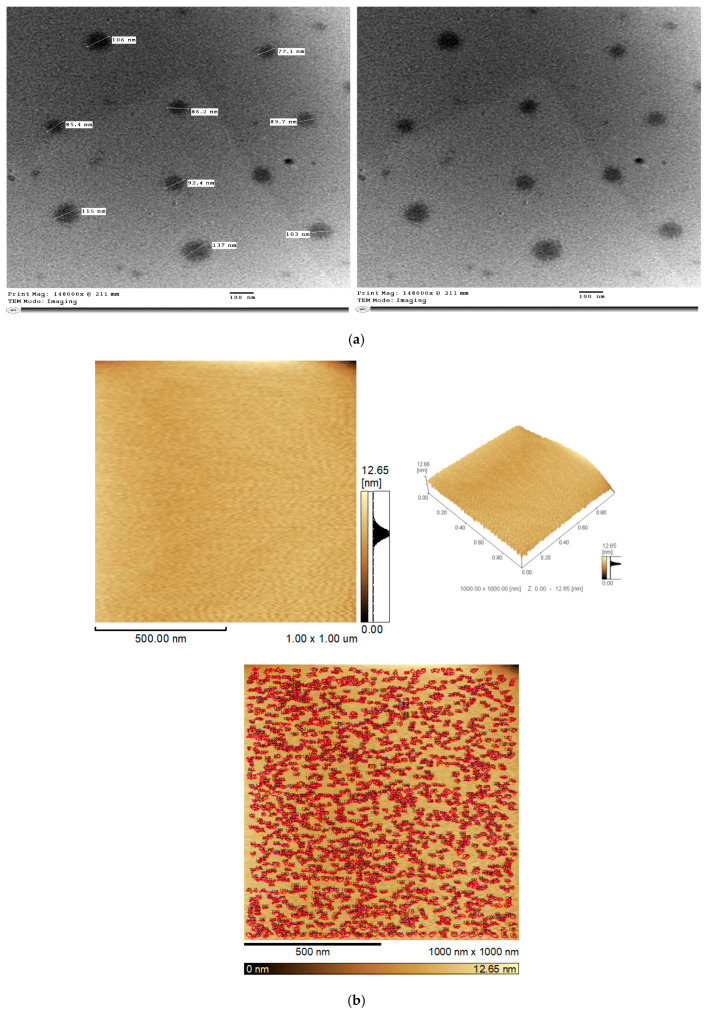
(**a**) Transmission electron micrograph (TEM) for the optimum BSP-PLGA-NPs. (**b**) Atomic force microscopy of the optimum BSP-PLGA-NPs (AFM).

TEM is a powerful tool for analyzing the morphology, dispersion, homogeneity, and internal structure of nanoparticles [99]. The ideal TEM micrograph displays well-defined, round particles with minimal aggregation. The morphometric properties of the optimum BSP-PLGA-NPs were assessed by TEM imaging. Figure 3a depicted spherical, smooth surfaces without any clumps or aggregations and the mean particle sizes were comparable to those mentioned before in the particle size investigations.

#### 3.2.2. Atomic Force Microscopy

Atomic force spectroscopy is a very potent tool for nanoscale surface analysis and characterization. AFM imaging is usually applied to scan the tip across a surface to visualize surface topography, which refers to a surface’s arrangement and features. It also provides valuable information about nanoparticles’ size, shape, and distribution. The optimized nanoparticles appeared smooth, homogenous, well-defined, and arranged as depicted in Figure 3b.

#### 3.2.3. Fourier Transformed Infrared Spectroscopy

**Figure 4:**
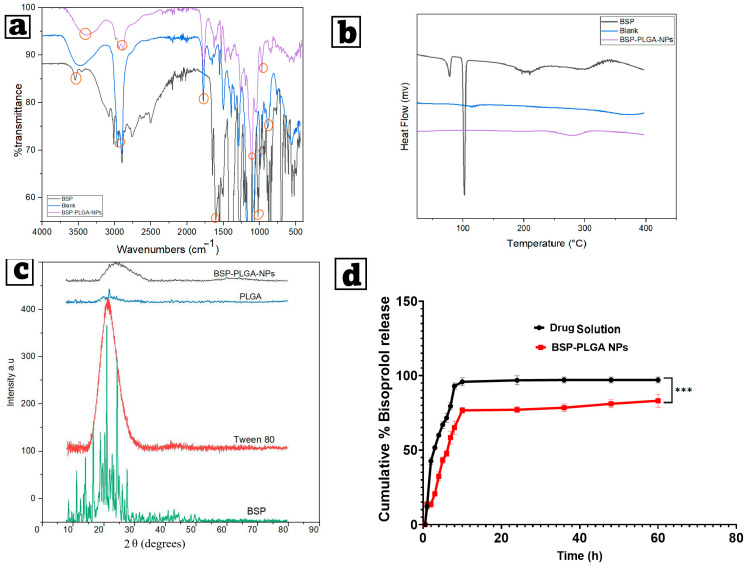
(**a**) Fourier transform-infrared spectroscopy examination (FT-IR), (**b**) differential scanning calorimetry thermograms (DSCs), (**c**) X-ray diffractogram (XRD) for BSP, blank, and chosen optimum BSP-PLGA-NPs, and (**d**) in vitro release patterns of the chosen optimum BSP-PLGA-NPs formula compared to drug suspension. Note: *** indicates significance between data with *p* value < 0.001.

FTIR was employed to analyze the chemical content and structure of the samples, which were revealed by the removal or broadening of peaks and alterations in the wave number, which may signify a complex formation. Figure 4a depicts the FTIR spectrum of pure bisoprolol, showing sharp peaks at 1085.61 cm^−1^ that are characteristic of stretching of (C–O) of secondary alcohol (propanol), at 1733.93 cm^−1^ that are characteristic of stretching of the carbonyl group (C=O), and at 3504.79 cm^−1^ that are characteristic of stretching of secondary amine. The poly lactic-co-glycolic acid showed peaks related to the stretching of the aliphatic C-H (2856.70 cm^−1^). The C=O ester (1746.43 cm^−1^), the carbonyl C=O stretch (847.90 cm^−1^), and the (C–O) aliphatic polyesters group were observed at 1092.73 cm^−1^. The FTIR spectrum of BSP-PLGA-NPs depicted a wide peak at 3384.03 cm^−1^ related to slight shifting in the peak of the secondary amine (N-H) of BSP, slight shifting of the secondary alcohol (propanol) of BSP at 1027.83 cm^−1^, and a sharp peak at 1090.40 cm^−1^ resulting from slight shifting of the C-O stretching of the ester linkage found in PLGA, which indicate that slight drug–polymer interactions might have happened.

#### 3.2.4. Differential Scanning Calorimetry

The drug’s physical condition within the nanoparticle structure significantly influences the release of the active ingredient from the polymeric matrix. DSC investigations were performed to evaluate the thermograms of BSP, BSP-PLGA-NPs, and their blank. The bisoprolol thermogram showed an endothermic sharp peak at 102.66 °C, which disappeared in the thermogram of the formula, thus indicating incorporation of the drug inside the nanoparticles. The blank thermogram showed an endothermic peak at 130.35 °C due to glass transition of the polymer and a peak at 387.33 °C indicating its thermal breakdown, while the DSC thermogram of BSP-PLGA-NPs showed an endothermic peak at 215.45 °C, which may result from drug–polymer interaction, and another peak at 299.60 °C indicating thermal degradation of the drug–polymer system. This reflects that some changes in the thermal stability of the nanoparticles matrix occurred as a result of the drug, as shown in Figure 4b.

#### 3.2.5. X-Ray Diffraction Spectroscopy

XRD is an analytical and physical technique that determines a material’s state by examining the magnitude and morphology of the peaks. Significant and obvious peaks reveal a high degree of crystallinity, whereas rounded, broad peaks mean an amorphous structure. Figure 4c depicts X-ray diffractograms of the pure bisoprolol, revealing distinct angles at 2θ of 11.111°, 13.981°, 16.522°, 20.914°, and 24.287°. While the polymer (PLGA) showed a distinct peak at the 2θ angle of 21.66 and that of Tween 80 exhibited a round peak, which revealed its amorphous structure. BSP-PLGA-NPs showed no distinct peaks except for a broad, round peak indicating the incorporation of the drug inside the polymeric matrix.

#### 3.2.6. In Vitro Release Analysis

In vitro release analysis is a trustworthy approach to assess and determine the in vivo formulations’ bioavailability. Drugs can be released from PLGA nanoparticles either by diffusing through the polymer matrix or by degrading and eroding the polymer [48,100]. Most researchers suggest that PLGA biodegradation into lactic acid and glycolic acid occurs by hydrolysis rather than enzymatic activity [101]. Assuming that drug release contributes to drug–polymer interactions, drug parameters such as molecular weight, log-P, and concentration are also critical [40,102].

In most cases, the release of hydrophilic molecules occurs via a porous pathway, whereas hydrophobic ones pass through the hydrophobic PLGA matrix [103]. Figure 4d illustrates the in vitro release investigations of BSP from both BSP solution and BSP-PLGA nanoparticles. The release of BSP from the solution occurred promptly, with around 50% being released during the initial 2 h and nearly 80% after 6 h. The release of BSP from BSP-PLGA-NPs exhibited an initial burst effect assigned to the release of unencapsulated BSP maintained on the particle surface. This initial burst occurs due to a rapid release of a substantial amount of the drug upon contact with the release medium, before a more sustained release begins. This frequently happens in PLGA-based systems and is often induced by hydrophilic medicines, allowing for fast diffusion. Some factors that boost burst release include polymer features, formulation parameters including the polymer concentration and surface properties, and medication properties, such as the drug’s solubility. Following an initial burst, a persistent release phenomenon was seen whereby the drug diffuses across the channels in the polymer, with more than 80% of the drug being released over 12 h, maintaining a long-term therapeutic level. It was noticed that a low initial burst effect and a more constant release profile complied with an increasing PLGA concentration. To further understand the release process, we used in vitro release data of BSP-PLGA-NPs from multiple empirical kinetic models (zero-order, first-order, Higuchi, and Korsmeyer-Peppas models) via nonlinear regression. The Korsmeyer-Peppas model had the strongest correlation coefficient (R^2^ = 0.987), which indicates that drug release occurs through a combination of diffusion and erosion from the PLGA matrix. The Korsmeyer-Peppas model provided the best match (R^2^ = 0.987), which indicates a diffusion-erosion process (*n* = 0.46), which is typical of non-Fickian transport in PLGA systems. This suggests that drug diffusion through hydrated polymer channels, as well as slow polymer degradation, contribute to the observed persistent biphasic release. Anomalous (non-Fickian) diffusion is characteristic of hydrophilic medicines placed inside a biodegradable polymer matrix.

### 3.3. Short-Term Stability of BSP-NPs

The optimized formula was kept fresh at 25 °C and 4 °C for three months; the values of the PS, PDI, ZP, and EE% are displayed in Table 3. After 3 months, a decrease in ZP and an increase in PS were observed. It is worth mentioning that polymeric nanoparticles have poor stability in aqueous suspension. Moreover, water removal from the formulations by lyophilization is recommended, as reconstituted freeze-dried samples are more suitable for ocular administration. References [104,105] obtained similar results when optimized PEGylated PLGA nanospheres were designed by the design of experiments for the ocular administration of dexibuprofen.

### 3.4. Ex-Vivo Analysis

#### Confocal Laser Scanning Microscopy Analysis (CLSM)

**Figure 5:**
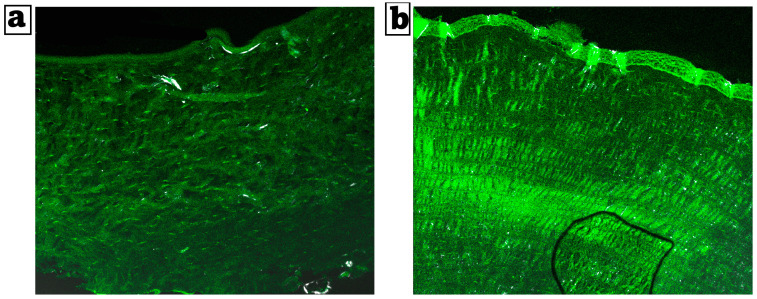
(**a**) Comparative CLSM pictures depicting the depth of cornea RhB-bisoprolol solution and (**b**) RhB-loaded BSP-PLGA-NPs. Magnification ×20.

Figure 5a,b shows the extent of nanoparticle infiltration into the cornea ocular tissues. The bisoprolol-loaded nanoparticles formula reached deeper down into the corneal layers, as demonstrated by uniformly dispersed fluorescence of high intensity. The tiny size of the nanoparticles, which offers an extensive surface area and the incorporation of the drug inside the PLGA polymer, clarified the noticeable substantial penetration of the formulation.

### 3.5. In Vivo Analysis

#### 3.5.1. Ocular Irritancy Test (Draize Test)

**Figure 6:**
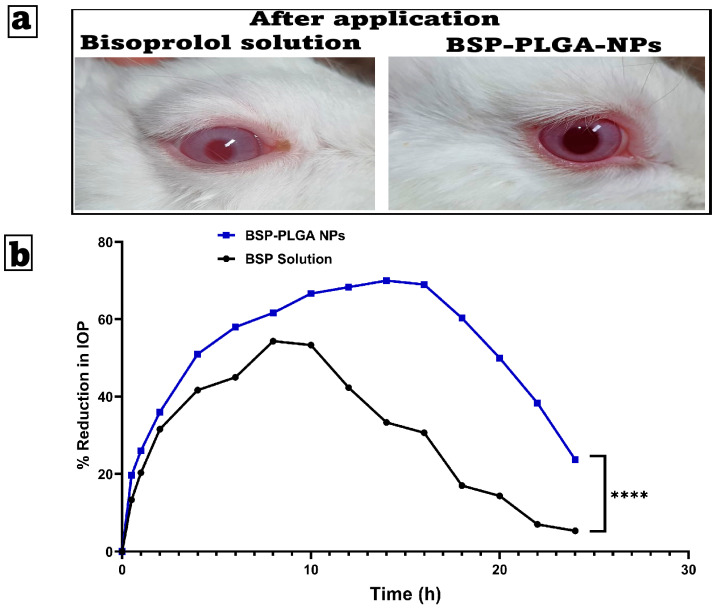
(**a**) Examination of possible ocular irritation following the application of bisoprolol solution and BSP-PLGA-NPs, and (**b**) the mean percentage decrease in intraocular pressure time profiles after an administration of a single ocular dose of bisoprolol solution (BSP Solution) and bisoprolol-loaded PLGA nanoparticles (BSP-PLGA NPs) in rabbit eyes (mean ± SE, *n* = 6, **** *p* < 0.0001).

The optimum formulation was placed into the left eye of three male albino rabbits and examined for 21 days. Scores on the Draize test range from 0 (no irritation) to 3 (maximum irritation and redness). Based on the level of irritation, we assumed that a score of zero of the optimum formulation was determined to be harmless and non-allergic. The bisoprolol solution showed slight irritation, while BSP-PLGA-NPs exhibited no irritation owing to drug encapsulation inside the polymeric matrix. The investigation discovered that no animal sustained visual harm throughout the mentioned duration. As a result, the optimized nanoparticles were non-irritating, safe, and suitable for prolonged ocular use, as shown in Figure 6a.

#### 3.5.2. Pharmacodynamic Analysis

Figure 6b shows the effect of BSP solution and BSP-PLGA NPs on the reduction in intraocular pressure (IOP) in rabbits over time. BSP-PLGA NPs have a greater and longer-lasting effect than the other formulations, but both cause an initial rapid decrease in intraocular pressure (IOP). After four to five hours, the BSP solution reaches a peak reduction of about 50% before beginning to decline. On the other hand, BSP-PLGA NPs achieve a higher peak of approximately 70% and maintain the reduction for a longer period, with significant effects lasting more than 10 h. Nanoparticles are responsible for this prolonged action, which can be attributed to the nanoparticles themselves. The intraocular pressure (IOP) reduction effects of two formulations, BSP-PLGA nanoparticles (NPs) and a BSP solution, over a duration of 24 h were observed. During the first four hours, both formulations reduce intraocular pressure rapidly, with the BSP solution showing a slightly faster beginning of its activity. By hour four, the BSP-PLGA nanoparticles have a more pronounced effect, surpassing the IOP drop achieved by the BSP solution. This pattern has been stable throughout time. The BSP solution produces a maximum intraocular pressure drop of approximately 55% during an 8 to 10 h period. The efficacy drops dramatically after this time, falling below 10% by the 24 h mark. The BSP-PLGA nanoparticles exhibit a prolonged therapeutic effect compared to alternatives. Their maximum effect approaches 70% at approximately 14 h and stays above 50% until 20 h, subsequently declining to about 25% by the conclusion of the observation period.

The nanoparticle-based controlled release, which improves ocular drug retention and bioavailability, takes responsibility for this. It would appear, based on the findings, that the BSP-PLGA NPs provide a more effective therapeutic effect in comparison to the BSP solution. This may lead to a decrease in the number of times that dosing is required, as well as an improvement in the results of treatment for glaucoma.

#### 3.5.3. Histopathological Examination

**Figure 7:**
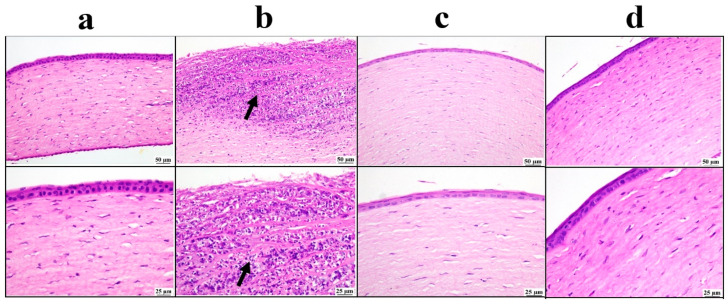
Photomicrographs of the cornea: (**a**) negative control group showing normal corneal structure, (**b**) positive control group showing ulceration with mononuclear inflammatory cells infiltration inside the cornea (arrow), (**c**) bisoprolol solution-treated group showing moderate restoration of cornea structure, (**d**) BSP-PLGA-NP-treated group showing marked restoration of normal structure of cornea with low and higher magnification (H&E).

Microscopic examination of the cornea (Figure 7) revealed a normal corneal structure in the negative control group. Meanwhile, the positive control group showed extensive corneal ulceration with heavy mononuclear inflammatory cell infiltration. Both the drug solution and nano groups showed marked improvement, as the examined corneal sections were apparently normal in structure.

**Figure 8:**
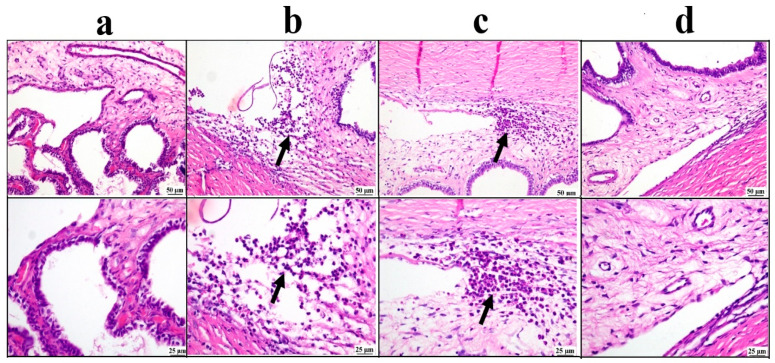
Photomicrographs of the ciliary body: (**a**) negative control group showing normal structure of ciliary body, (**b**) positive control group showing inflammatory edema at the filtration angle and ciliary body (arrow), (**c**) bisoprolol solution-treated group showing localized aggregation of inflammatory cells (arrow), (**d**) BSP-PLGA-NP-treated group showing marked restoration of the normal structure of ciliary body with low and higher magnification (H&E).

The ciliary body sections examined from the negative control group showed a normal structure of the ciliary body. On the contrary, the positive control group exhibited relevant edema with inflammatory cell infiltrations. The drug solution group showed moderate improvement, as the examined sections revealed focal aggregation of inflammatory cells. The BSP-PLGA-NPs group showed marked improvement, and apparently restored the normal structure of the ciliary body (Figure 8).

**Figure 9:**
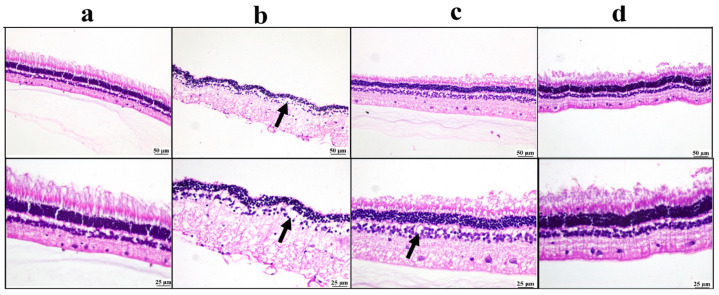
Photomicrographs of the retina: (**a**) negative control group showing normal retinal structure, (**b**) positive control group showing marked edema with a reduction in retinal neurons (arrow), (**c**) bisoprolol solution-treated group showing mild retinal edema (arrow), (**d**) BSP-PLGA-NP-treated group showing marked restoration of normal retinal structure with low and higher magnification (H&E).

The negative control group showed a normal appearance of the retinal structure. The positive control group showed marked retinal edema with reduced retinal cells. Mild improvement was detected in the drug solution group, as there was mild edema; meanwhile, the BSP-PLGA-NPs group showed marked restoration of the normal retinal structure (Figure 9).

### 3.6. Immuno-Histochemistry (IHC)

Glial fibrillary acidic protein (GFAP) is a major marker found in Müller retinal cells. GFAP expressions are measured using immunohistochemistry in a glaucoma rabbit model. In glaucomatous eyes’, the retinas would probably exhibit altered astrocyte morphology and elevated GFAP expression, and this would be in line with how neuroinflammation and astrocyte activation contribute to the development of glaucoma.

**Figure 10:**
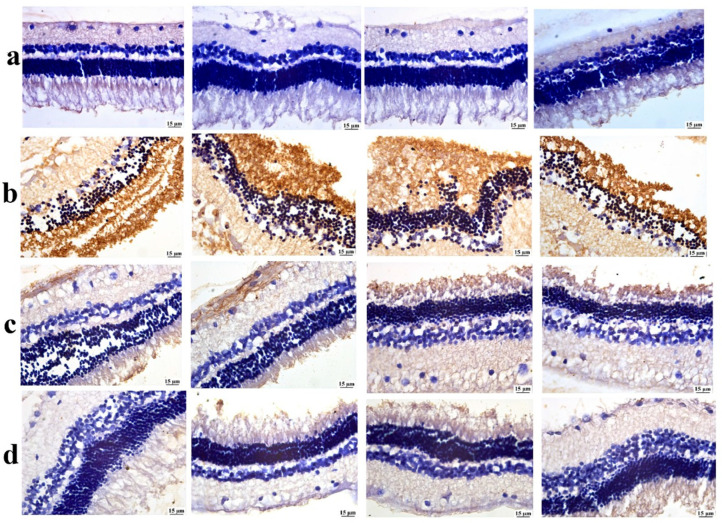
Photomicrographs of the retina, (**a**) the negative control group showing limited GFAP expression, (**b**) the positive control group showing intense GFAP expression, (**c**) the bisoprolol solution-treated group showing moderate GFAP expression, and (**d**) the BSP-PLGA-NP-treated group showing mild GFAP expression (immune staining).

As illustrated in Figure 10, the positive control group revealed a significant elevation in GFAP expression compared with the negative control group. Both treated groups showed a significant reduction in retinal GFAP expression compared to the positive control group. Among both treatments, the lowest GFAP value was detected in the BSP-PLGA-NP-treated group.

**Figure 11:**
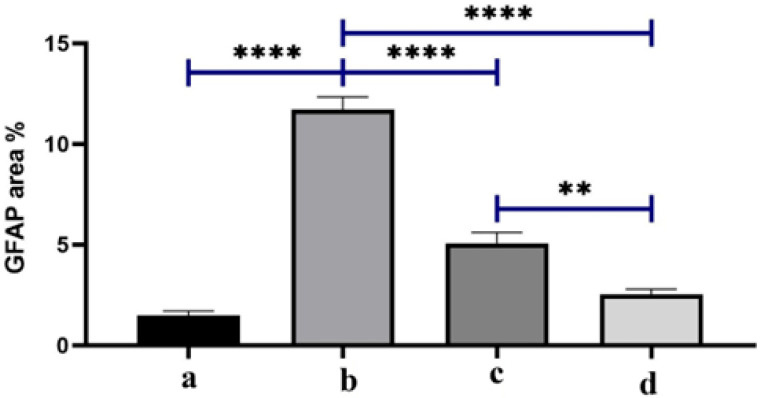
Quantification of GFAP as area percentage. Note: (a) the negative control group, (b) the positive control group, (c) the bisoprolol solution-treated group, and (d) the BSP-PLGA-NP-treated group. Data are expressed as the mean ± SE (*n* = 6): ** *p* < 0.01 and **** *p* < 0.0001.

Figure 11 represents the quantification of GFAP as an area percentage The positive control (b) shows the largest GFAP area %, which means that dexamethasone induction really activates glia in glaucomatous eyes compared to baseline (a). This supports the concept that steroid-induced ocular hypertension enhances neuroinflammatory signaling and gliosis. The group treated by BSP solution (c) shows a big drop in GFAP compared to b (*p* < 0.0001, brackets ****). This suggests that topical bisoprolol reduces glial activation, possibly by lowering intraocular pressure and providing neuroprotection through β1-blockers, as shown in previous studies. BSP-PLGA-NPs (d) show the lowest GFAP levels of all the treated groups, which is a lot lower than both b and c, which means that the nanoparticle system has a stronger anti-gliotic impact. Mechanistically, this is consistent with enhanced ocular penetration and sustained release, which we demonstrated through the CLSM depth of penetration, in-vitro release, and prolonged IOP reduction versus solution, and which thereby reduce the glial activation stimulus over time. Together, the data indicate that nanoparticle delivery augments bisoprolol’s biological effect on retinal glia, beyond what is achieved by the drug solution. We attribute this to the sub-200 nm hydrodynamic size aiding corneal passage, stabilizer-assisted dispersion with sufficient colloidal stability, and sustained intraocular concentrations, which were demonstrated functionally by longer-lasting IOP lowering, all of which reduce mechanical/ischemic stress that drives glial reactivity. The GFAP area % was analyzed by one-way ANOVA with Tukey post-hoc across all four groups; brackets show pairwise significance (** *p* < 0.01; **** *p* < 0.0001).

## 4. Conclusions

In our presented study, bisoprolol-PLGA loaded nanoparticles were optimally formulated by the solvent displacement approach. The formulations’ response illustrated the influence of several variables on the responses. The statistical findings demonstrated that the concentrations of the drug, PLGA, and Tween 80 influenced the entrapment efficiency %, zeta potential, particle size, and drug release from the polymeric matrix of nanoparticles. The zeta potential values (−18 mV) signified a stable nano system. TEM micrographs revealed the formation of rounded nanosized bisoprolol-loaded nanoparticles. The FTIR investigation examined perfect drug encapsulation inside the nanoparticles. The drug release from the nanoparticles seemed to be biphasic. The formulation exhibited a primary fast release due to the drug adsorbed on the PLGA nanoparticles’ surface, preceded by a prolonged release of more than 80% of the drug within almost 12 h. Confocal laser scanning microscopy confirmed deep penetration of the optimized formula inside the corneal tissue. Furthermore, the nano formulation superiorly lowered the intraocular pressure compared to the BSP solution, maintaining prolonged release of the drug and improving its permeability and bioavailability. Bisoprolol-loaded PLGA nanoparticles are a promising system for topical administration for glaucoma management. In vivo, the formulation was non-irritating in the Draize test, produced a greater and longer-lasting % reduction in IOP versus BSP solution, and improved ocular histopathology, restoring near-normal architecture of the cornea, ciliary body, and retina in the dexamethasone model. Overall, our findings suggest the increased ocular delivery and pharmacodynamic efficacy of BSP-PLGA nanoparticles for topical glaucoma therapy. However, the GFAP data show a connection rather than a direct mechanistic suppression of glial pathways; more research including multiple markers and pathway analyses is needed to prove causality.

## Figures and Tables

**Table 1:** Factorial design employed for the optimization of BSP-loaded PLGA nanoparticle formulations.

Factors (Independent Variables)	Design Levels		
	Low (−1)	Medium (0)	High (+1)
X1: Bisoprolol (mg)	12.5	25	37.5
X2: PLGA (mg)	22.5	25	27.5
X3: Tween 80 (mg)	60	80	100
Responses (Dependent variables)	Goal		
Y1: P.S (nm)	Minimize		
Y2: P.D.I (nm)	Minimize		
Y3: Z.P (mV)	Maximize		
Y4: E.E (%)	Maximize		

Abbreviations: BSP, bisoprolol; P.S, particle size; PDI, polydispersity index; ZP, zeta potential; and E.E, entrapment efficiency.

**Table 2:** Presents the composition of the variably manufactured BSP-loaded nanoparticles, in addition to the corresponding values of response variables for the determination of the optimal formulation (*n* = 3 ± SE).

Factors				Responses			
Run	A: Bisoprolol (mg)	B: PLGA (mg)	C: Tween 80 (mg)	PS (nm)	PDI	ZP (mv)	EE%
1	37.5	25	60	503 ± 0.32	0.49 ± 0.12	−29.3 ± 0.31	80 ± 0.46
2	12.5	27.5	100	179 ± 0.24	0.221 ± 0.01	−21.8 ± 0.45	50 ± 0.98
3	12.5	27.5	60	140 ± 0.18	0.22 ± 0.02	−21.8 ± 0.32	55 ± 0.72
4	12.5	22.5	100	140 ± 0.52	0.24 ± 0.02	−13.3 ± 0.51	65 ± 0.76
5	25	25	100	384 ± 0.71	0.96 ± 0.05	−18.6 ± 0.14	45 ± 0.88
6	12.5	25	80	389 ± 0.42	0.39 ± 0.01	−22.9 ± 0.52	60 ± 0.35
7	37.5	25	100	636 ± 0.31	0.69 ± 0.03	−12 ± 0.60	85 ± 0.65
8	25	25	100	784 ± 0.71	0.96 ± 0.05	−18.6 ± 0.14	45 ± 0.35
**9**	**25**	**22.5**	**60**	**105** ± **0.35**	**0.411** ± **0.14**	**−18.7** ± **0.41**	**75 ± 0.98**
10	12.5	25	80	389 ± 0.53	0.39 ± 0.11	−22.9 ± 0.61	60 ± 0.34
11	37.5	27.5	100	195 ± 0.62	0.127 ± 0.04	−30.5 ± 0.80	40 ± 0.56
12	37.5	22.5	80	147 ± 0.15	0.34 ± 0.03	−21.5 ± 0.47	70 ± 0.82
13	25	27.5	80	484 ± 0.50	0.96 ± 0.04	−18.6 ± 0.38	45 ± 0.47
14	37.5	27.5	60	503 ± 0.32	0.49 ± 0.06	−29.3 ± 0.31	80 ± 0.37
15	12.5	22.5	80	140 ± 0.87	0.24 ± 0.04	−13.3 ± 0.16	65 ± 0.72
16	25	27.5	80	484 ± 0.53	0.96 ± 0.04	−18.6 ± 0.38	45 ± 0.85

Abbreviations: BSP, bisoprolol; P.S, particle size; PDI, polydispersity index; ZP, zeta potential; and E.E, entrapment efficiency. Run 9 (bold): optimized formula.

**Table 3:** The short-term stability results of optimized bisoprolol hemifumarate-loaded PLGA nanoparticles at 4 °C and 25 °C for 3 months. Mean ± SE (*n* = 3).

Parameters	BSP-PLGA-NPs Freshly Prepared	BSP-PLGA-NPs After Three Months of Storage at 4 °C	BSP-PLGA-NPs After Three Months of Storage at 25 °C
**PS (nm)**	105 ± 0.35	140 ± 0.73	164 ± 0.51
**PDI**	0.411 ± 0.14	0.435 ± 0.12	0.487 ± 0.11
**ZP (mV)**	−18.7 ± 0.41	−16 ± 0.021	14 ± 0.03
**EE (%)**	75 ± 0.98	71 ± 0.06	69 ± 0.91

## Data Availability

The manuscript contains all pertinent data; any extra necessary documents are accessible upon request.

## Abbreviations

The following abbreviations are used in this manuscript:

EE%	entrapment efficiency
PS	particle size
PDI	polydispersity index
ZP	zeta potential
O.F	optimized formula
NPs	nanoparticles
PLGA	poly lactic co -glycolic acid
BSP	bisoprolol
DSC	differential scanning calorimetry
IHC	immuno-histochemistry
TEM	transmission electron micrograph
AFM	atomic force microscopy
FTIR	Fourier transform infrared spectroscopy
XRD	X-ray diffraction
CLSM	confocal laser scanning microscopy
GFAP	glial fibrillary acidic protein
